# Recent Advances in Mono- and Combined Stem Cell Therapies of Stroke in Animal Models and Humans

**DOI:** 10.3390/ijms20236029

**Published:** 2019-11-29

**Authors:** Roxana Surugiu, Andrei Olaru, Dirk M. Hermann, Daniela Glavan, Bogdan Catalin, Aurel Popa-Wagner

**Affiliations:** 1 Center of Clinical and Experimental Medicine, University of Medicine and Pharmacy, 20049 Craiova, Romania; roxana.surugiu07@gmail.com; 2 Department of Ophthalmology, University of Medicine and Pharmacy, 20049 Craiova, Romania; andrei8317@gmail.com; 3 Chair of Vascular Neurology, Dementia and Ageing Research, Department of Neurology, University of Duisburg-Essen, University Hospital Essen, 45122 Essen, Germany; dirk.hermann@uk-essen.de; 4 Department of Psychiatry, University of Medicine and Pharmacy, 20049 Craiova, Romania; danaglavan@gmail.com; 5 Experimental Research Centre for Normal and Pathological Aging, University of Medicine and Pharmacy of Craiova, 20049 Craiova, Romania; 6 Griffith University Menzies Health Institute of Queensland, Gold Coast Campus and Queensland Eye Institute, Brisbane QLD 4000, Australia

**Keywords:** human stem cells, rodent stem cells, cerebral ischemia, aging, restorative therapies

## Abstract

Following the failure of acute neuroprotection therapies, major efforts are currently made worldwide to promote neurological recovery and brain plasticity in the subacute and post-acute phases of stroke. Currently, there is hope that stroke recovery might be promoted by cell-based therapies. The field of stem cell therapy for cerebral ischemia has made significant progress in the last five years. A variety of stem cells have been tested in animal models and humans including adipose stem cells, human umbilical cord blood-derived mesenchymal stem cells, human amnion epithelial cells, human placenta amniotic membrane-derived mesenchymal stem cells, adult human pluripotent-like olfactory stem cells, human bone marrow endothelial progenitor cells, electrically-stimulated human neuronal progenitor cells, or induced pluripotent stem cells (iPSCs) of human origin. Combination therapies in animal models include a mix of two or more therapeutic factors consisting of bone marrow stromal cells, exercise and thyroid hormones, endothelial progenitor cells overexpressing the chemokine CXCL12. Mechanisms underlying the beneficial effects of transplanted cells include the “bystander” effects, paracrine mechanisms, or extracellular vesicles-mediated restorative effects. Mitochondria transfer also appears to be a powerful strategy for regenerative processes. Studies in humans are currently limited to a small number of studies using autologous stem cells mainly aimed to assess tolerability and side-effects of human stem cells in the clinic.

## 1. Introduction

Cerebral ischemia is a metabolic disease of the blood vessels affecting mostly the older population for which no rehabilitative therapy exists. Therefore, worldwide there is a huge investment in research aimed at alleviating the sequelae after stroke and improving restorative recovery. Unlike other species, the human brain is extremely sensitive to lack of oxygen which causes within minutes neuronal death [1,2]. In addition, the older brains are refractory to neuronal regeneration, probably because of the inhibitory environment which does not allow for axonal reconstruction. For this and other reasons, efforts are currently made to promote alternative therapies including physical, pharmacological, or cell-based therapies some of which have been tested in small clinical trials [3]. Currently, there is hope that stroke recovery might be promoted by cell-based therapies. In this review, we focus on stem cells therapy for cerebral ischemia which has made significant progress in the last five years. A variety of stem cells have been tested in animal models and humans including adipose stem cells, human umbilical cord blood-derived mesenchymal stem cells, human placenta amniotic membrane-derived mesenchymal stem cells, induced pluripotent stem cells (iPSCs) of human origin. Emerging stem cells for stroke therapy include, human amnion epithelial cells, adult human pluripotent-like olfactory stem cells, human bone marrow endothelial progenitor cells, electrically-stimulated human neuronal progenitor cells.

## 2. Stem Cell Monotherapies in Animal Models

### 2.1. Human Placenta Amniotic Membrane-Derived Mesenchymal Stem Cells

Human placenta amniotic membrane-derived mesenchymal stem cells (AMSC) regulate the immune response. Under hypoxic conditions, the gene expression of several factors involved in the regulation of immunity is shifted. CD200, an anti-inflammatory factor and a TGF-β positive regulator, was more strongly expressed under hypoxic conditions than under normotoxic conditions. AMSC transplanted in the rat ischemic stroke model inhibited the expression of proinflammatory cytokines and increased CD200 levels compared to control group. In addition, rats treated with AMSC have shown low activation of microglia in the penumbra and improved behaviour. The results suggest that CD200 strongly expressed from AMSC in a hypoxic environment modulates inflammatory cytokine levels and microglia activation, thus increasing the therapeutic recovery potential after hypoxic-ischemic brain lesions, demonstrating the immunomodulatory function of AMSC in a stroke model [4].

In another study, human pluripotent stem cells with the potential to differentiate into cortical neurons, were evaluated upon transplantation in an animal model of ischemia in rats. Grafting at the lesion demonstrated reactivity to the margin of ischaemia, indicated by preferential axonal growth and cell migration, without influencing cell survival. Behaviourally, limb asymmetry was improved in the treatment group as compared to the control group [5].

### 2.2. Human Amnion Epithelial Cells (hAECs)

Human amnion epithelial cells (hAECs) are non-immunogenic, non-tumorigenic, anti-inflammatory cells normally discarded with the placenta. These biological features, broad availability and lack of ethical barriers in their use could make them useful as a therapy for ischemic stroke. The effectiveness of intravenous injection in the acute phase (1.5 h) or post-stroke (1–3 days) of the cells was tested in 4 animal models of cerebral ischemia, in young and old mice of both sexes, 7–14 weeks and 20–22 months as well as adult marmosets of either sex. hAECs administered at 1.5 h after stroke in mice migrated to the ischemic area via a CXC chemokine receptor type 4 dependent mechanism. The beneficial effects included reduced inflammation, infarct volume and functional deficits. Improved functional recovery was reported in young and elderly mice of both sexes even if hAEC administration was delayed up to 1 or 3 days after stroke. Intravenous injection of hAECs during the acute phase in marmosets led to a reduction in the infarct size. Systemic administration of hAECs determines marked neuroprotection and facilitates repair and recovery mechanisms [6].

### 2.3. Human Umbilical Cord Blood-Derived Mesenchymal Stem Cells (hUCB-MSCs)

The protective effect of human umbilical cord blood-derived mesenchymal stem cells (hUCB-MSCs) has been tested by administering hUCB-MSCs (0.25 million cells/animal and 1 million cells/animal) intravenously, through the tail vein, in male Sprague–Dawley rats that were subjected to a transient cerebral artery occlusion. Interestingly, hUCB-MSCs treatment prevented the induction of matrix metalloproteinases (MMPs), which were overexpressed in the control group, at both dosages. HUCB-MSCs treatment also prevented the induction of tissue inhibitors of metalloproteinases [7].

### 2.4. Exogenous Human Neuronal Progenitor Cells

Conductive polymers have been recently used for cell delivery, drug release, and electrical stimulation to improve post-stroke recovery. Thus, an electrically conductive polymer scaffold was used to induce changes in the expression of genes involved in cell survival, inflammatory response, and synaptic remodelling in human neuronal progenitor cells (hNPCs). hNPCs are a promising alternative for stroke treatment, but optimum delivery conditions are not known. Electrical stimulation of hNPC using conductive polymers was performed the day before implantation, and the cells were transplanted in a MCAo rat model. Electrochemically manipulated pre-conditioned hNPCs reportedly improved functional results as compared to unstimulated hNPCs [8,9]. However, it is not clear how to use electrical stimulation to make changes in gene expression that would favour neural tissue recovery. 

### 2.5. Human Bone Marrow Endothelial Progenitor Cells to Repair BBB

Blood brain barrier (BBB) impairment is a pathological manifestation secondary to ischemic stroke, being one of the targets of new stroke recovery therapies. Using electron microscopy, the ability of bone marrow endothelial progenitor cells (hBMEPCs) to repair BBB in adult Sprague–Dawley rats subjected to transient MCAo) was evaluated. hBMEPCs pre-labelled with β-galactosidase were injected intravenously 48 h after MCAo. Ultrastructural analysis of microvessels in rats with stroke in the control group revealed the typical pathology of BBB at 5 days post-transplantation. In contrast, in the hBMEPCs treated group the animals showed vigorous repair of the network of the blood vessels the striatum and motor cortex without detectable perivascular edema. The transplanted endothelial cells displayed a normal morphology with intact mitochondria [10].

### 2.6. Adult Human Pluripotent-Like Olfactory Stem Cells 

Adult human olfactory mucosa, a highly regenerative tissue, is a mosaic of stem cell population that can be used for therapeutic purposes. Adult pluripotent-like olfactory stem cells (APOSCs) have the ability of self-renewal and differentiate into all three germinal cell lines. A subpopulation of adult pluripotent-like olfactory stem cells are modulated by an epigenetic repressor of CBX7, which is essential for the maintenance of embryonic stem cells by preventing cellular senescence. Unlike APOSCs from CBX7 −/− mice, the CBX7 +/+ mice APOSCs respond to induced tissue regeneration or induced lesions. Quite interestingly, in vitro cultured CBX7 −/− APOSCs suffered from premature senescence, while CBX7 +/+ APOSC is actively dividing, indicating that CBX7 is required for self-renewal of APOSCs. Intracerebral implantation of APOSCs, obtained from biopsy specimens of human olfactory mucosa and tranduced with the Luc gene; migrated to the injury site; differentiated into neuronal, glial, endothelial cell types; and improved neurological stroke-mediated function in mice subjected to MCAo [11].

### 2.7. Induced Pluripotent Stem Cells (iPSCs) of Human Origin

Induced pluripotent stem cells (iPSCs) are derived from differentiated cells by transfection with transcription factors which can induce pluripotency. iPSCs are a good alternative as replacement for neural stem/progenitor cells (NSCs/NPs). In addition, iPS of human origin can be easily tracked in animal studies. Thus, human induced pluripotent neural stem cells (hiNSC BC19) transplanted into a pig model of stroke correlated with good recovery of brain integrity and perfusion, as evaluated by magnetic resonance imaging (MRI). In vitro hiNSCs expressed Nestin and Sox1 and differentiated into neurons, astrocytes, and oligodendrocytes. In vivo, iNSCs had a 12-week survival time and differentiated into oligodendrocytes and neurons, conferring neuroprotection by decreasing microglial activation and stimulating neurogenesis [12]. 

Quite recently, intra-parenchymal injection of human neuronal progenitor cells (hNPCs) in a model of stroke in pig, was associated with better functional recovery. The animals were divided into a treatment and a control group; the treatment group received intra-parenchymal stem cell injections on day 5 post stroke while the control group received injections of phosphate buffer saline (PBS). Animals were followed on weeks 1–12 post injection. Animals in the treatment group showed a quicker functional recovery and better preservation of appetite and posture as compared to the control group [13].

### 2.8. Studies Using Genetically Modified Multipotent Mesenchymal Stem Cells in Animal Models

MSCs exposed to extracts from ischemic brains seem to secrete a wide range of neuroprotective proteins including brain derived neurotrophic factor (BDNF), nerve growth factor (NGF), vascular endothelial growth factor (VEGF), and hepatocyte growth factor (HGF) [14]. Therefore, the use of genetic modified MSCs to release neurotrophic factors in the lesioned area is of particular interest. For a recent review on the use of genetically modified MSCs for the treatment of various diseases, including cardiovascular diseases and neurodegenerative, diseases, see [15]. Thus, Wyse and colleagues showed that transplantation of BM-MSCs that are modified using a retrovirus to overexpress NGF or BDNF into the striatum of a mouse model of Huntington disease led to improved behavioural and histological indices in treated animals over controls [16]. Similarly, autologous fibroblasts, engineered to express NGF, have been used to improve Mini-Mental Status Examination scores in patients with Alzheimers‘ disease [17]. Likewise, injection of autologous BM-MSCs expressing glial derived neurotrophic factor (GDNF) into the lateral ventricle of several patients with Parkinson disease led to beneficial results [18].

Endothelial progenitor cells (EPCs) along with the chemokine CXCL12 have the advantages of both promoting angiogenesis and would promote neurogenesis brain in part by secreting trophic factors. Much like G-CSF, the chemokine CXCL12 has been shown to attract EPCs, neural progenitor cells, and oligodendrocyte progenitor cells (OPCs) to the injury site. By using a lentivirus overexpressing CXCL12 in human endothelial progenitor cells (hEPC-cxcl12), it was reported on synergistic effects of the combination therapy over monotherapies by stimulating angiogenesis, neurogenesis, and migration of oligodendrocyte progenitor cells to the lesion site. Increased vessel density and preservation of the myelin sheath integrity in the treatment groups were also reported [19].

In a recent study, the authors hypothesized that restoration of the interhemispheric connectivity of the motor cortex might be an additional functional recovery mechanism. Using imaging methods (MRI diffusion tensor imaging) and neuroanatomical tracing techniques by injecting a GFP-expressing adeno-associated (AAV) virus, an anatomical restoration of interhemispheric connections through the corpus callosum could be demonstrated following intravenous MSC administration of autologous cells in a rat model of stroke (MCAo). Moreover, the degree of connectivity was higher in the MSC treated group than in the vehicle-treated group. Accordingly, both the thickness of corpus callosum and synaptic puncta in the contralateral motor cortex were greater in the MSC treated group than in the control group [20].

### 2.9. Adipose Stem Cells 

Recently, adipose tissue-derived mesenchymal stem cells (ADMSCs) have demonstrated their therapeutic potential in stroke models by improving functional recovery, with an increase in markers related to brain repair in animal models of stroke. Thus, adipose stem cells (ASCs) labelled with superparamagnetic iron oxide particles (VSOP), which allow cell tracking by MRI, were given intra-arterially to a rat model of stroke, and an MRI analysis was performed at 48 h and 9 days after MCAo. An increased ASCs signal strength in the affected hemisphere at 48 h post MCAo was indicative of an active migration of ASCs to the infarction site which was proportional with the number of injected cells. However, the infarct volume was increased after injection of 1 × 10^6^ ASC as compared to controls or the group treated with 5 injections of 10^4^ ASCs. At 9 days after MCAo, injection of 3 × 10^5^ ASCs resulted in a reduced infarct volume. It was concluded that intra-arterial administration is an efficient method, but its usefulness greatly depends on the proper dose of delivered stem cells, as this factor has a strong influence on the migration rate and infarct volume, with better outcomes at the lower doses [21].

Quite recently, an adipose tissue stem cell line RESSTORE01, has been used to improve functional recovery in adult male Sprague–Dawley rats subjected to permanent occlusion of the MCA. After the intravenous infusion of vehicle or 2 × 106 ADMSCs behavioural recovery was assessed by Rogers’ functional evaluation scale, which assess reflexes, sensory responses, and simple motor functions and performance in the cylinder and sticky label tests during a 42-day behavioural follow-up. Tissue recovery was assessed using histological markers of angiogenesis (RECA-1), gliosis (GFAP), and glial scar formation [22].

### 2.10. Bone Marrow Stromal Cells

Bone marrow stromal cells (BMSC) give rise to various non-hematopoietic cells including osteoblasts and adipocytes and are normally used for treating bone-related abnormalities. Hypoxia is essential in the mobilization and homing of MSCs, by its ability to induce stromal cell-derived factor-1 expression along with its receptor, CXCR4. For this reason, transplantation of BMSC holds the promise for ischemic stroke therapy. Within the hypoxic environment, BMSC secrete vascular endothelial growth factor and interleukin-6, thereby promoting wound healing and diabetic fracture healing. A recent study investigated the hypothesis that hypoxic preconditioning could increase the effectiveness of BMSC transplantation in a rat model of ischemic stroke. Post-stroke rats received normally cultured bone marrow stromal cells (N-BMSCs), BMSCs exposed to hypoxia (H-BMSCs), or DMEM cell culture medium (control) at 24 h after cerebral ischemia. Compared to the control group, the H-BMSCs-transplanted group exhibited significantly improved functional recovery and diminished infarct volumes over the N-BMSCs- and DMEM-transplanted groups. The increased survival and differentiation of transplanted cells and improved neurological function involved the inhibition of caspase-3 activation and an increased expression of HIF-1α, which is indispensable for angiogenesis and neurogenesis [23,24].

### 2.11. Combination Therapies in Animal Models

It is unlikely that monotherapies will address the tissue and functional recovery after cerebral ischemia. Rather, a monotherapy might be helpful at a certain stage of post-stroke recovery. Therefore, it is reasonable that combination therapies could be more efficacious at multiple time points of tissue and functional recovery after cerebral ischemia. Our group was interested in the potentially beneficial effect of combination therapies using bone marrow derived mesenchymal stem cells (BM-MSCs) and bone marrow derived mononuclear cells (BM-MNCs). Intrajugular administration of a single dose (1 × 10^6^) of such stem cells alone or in combination with the chemoattractant, Granulocyte Colony Stimulating Factor (G-CSF) at 3 hrs after cerebral ischemia in aged rats led to some improvement of some behavioural indices of recuperation at 48 days after the event [25,26]. Further, the combination therapy increased neurogenesis in the subventricular zone and improved microvessel density in the formerly infarct core and perilesional area of treated aged rats. However, the combined treatment had no beneficial effect on the mortality or infarct volume.

In clinical practice, the efficacy of cell-based therapies in patients can be improved if combined with an enriched environment. Indeed, the combination of human adipose tissue-derived mesenchymal stem cells (ADMSCs) with enriched environment was superior to the monotherapy alone [22].

### 2.12. The Combined Effect of Bone Marrow Stromal Cells, Exercise, and Thyroid Hormones

A recent study evaluated the combined effect of bone marrow stromal cells (BMSC) administration with exercise and administration of thyroid hormones in a model of middle cerebral artery occlusion (MCAO) in young mice. The stem cells were injected into the right ventricle at 24 h after MCAO, followed by daily T3 injections and physical training for 6 days. Animals were perfused on day 7 after stroke. Functional recovery, infarct volume, and the number of BrdU and TUNEL-positive cells were evaluated. All combination therapies led to a decrease in the infarct volume: BMSCs and exercise by 51%, BMSCs and T3 by 26%, BMSCs + T3 and exercise by 70%. A significant decrease in the infarct volume (by 26%) was also seen in the BMCS monotherapy as well as the exercise alone group, by 44%. A functional improvement on neurobehavioral tests was observed in the exercise groups, BMSCs and Physical Exercise and the full combination, BMSC + T3 and Physical Exercise. Combination therapy also led to a decrease in the number of TUNEL cells, which suggests a decrease in apoptosis, and an increase in the number of BrdU-positive cells [27]. An overview of current stem cell therapies for cerebral ischemia is shown in Table 1.

### 2.13. Autologous Stem Cell Monotherapies in Humans

A prospective study involving a small cohort of 20 stroke patients, divided into 2 groups, intervention and control, assessed the safety and clinical outcome after treatment with autologous stem cells in the subacute stage of ischemic stroke. Autologous stem cells were given intra-arterially to the ipsilateral MCA of subacute ischemic stroke patients. Patients in the intervention group received intra-arterial stem cell infusion in the ipsilateral middle cerebral artery on days 8–15 post-stroke. Final analysis at 6 months was performed for primary (safety) and secondary outcomes (efficacy). No complications were reported after intra-arterial injection, and procedure-related mortality was not an issue for both groups. The clinical response using the modified Rankin scale was superior for patients in the treatment group. Intra-arterial infusion of stem cells can be carried out safely in the subacute stage of ischemic stroke. Improved clinical outcomes were observed with intra-arterial stem cell therapy [28].

Another prospective pilot trial involved a small group of 12 patients with a history of stroke (from 3 months to 2 years). Patients were allocated to two groups, those who received intravenous autologous ex vivo cultured mesenchymal stem cells (MSC group) or those who did not (control group), and all were followed for four years from the day of cell transplantation. Patients were followed for 4 years, with regular assessment of muscle strength on the upper limbs. The transplanted cells were well tolerated. There were no cell-related side effects and all patients survived. Moreover, a modified Barthel Index showed a statistically significant improvement at 156 and 208 weeks of transplantation in the MSC group as compared to controls. The results of the study indicated some potential for the use of autologous mesenchymal stem cells for stroke patients to improve functional recovery [29].

Adult pluripotent-like olfactory stem cells (APOSCs) have the ability of self-renewal and differentiate into all three germinal cell lines. In a small clinical trial autologous APOSCs were injected to six patients under stereotactic guidance and were followed up to 3 mo. Patients in the treatment group seemed to present better clinical outcomes than those with low expression of CBX7 in their autologous APOSCs. Overall, despite a low differentiation efficiency into neuronal cells, these findings indicate the safety and feasibility of autologous APOSC implantation in the treatment of stroke [11].

### 2.14. Mechanisms Underyling the Beneficial Effects of Transplanted MSCs

During the early stages of cell therapy, it was hypothesised that MSCs contribute to tissue regeneration by replacement of lost cells. However, MSCs cannot survive for long at the site of transplantation and do not generate functional neurons to replace the lost ones. Therefore, focus has shifted to their paracrine actions or the so called the ‘‘bystander’’ effect. A common hypothesis is that the beneficial effects of transplanted MSCs are mediated either by “chaperones” or through paracrine mechanisms [30]. Cell-based therapy and for tissue regeneration is based on MSC capability to produce a large variety of trophic factors and chemokines that stimulate neighbouring parenchymal cells to start repairing damaged tissues [31].

Other mechanisms include the preservation of the blood brain barrier (BBB) integrity. Ischemic stroke leads to increased MMP-9 (matrixmetalloproteinase-9) levels, causing the breakdown the blood brain barrier (BBB). The hypothesis of this study was that mesenchymal stem cells protect the blood-brain barrier by inhibiting MMP-9 through a mechanism that involves intercellular adhesion molecule-1 (ICAM-1). Treatment of post-stroke male adult IRC mice with 2 × 105 MSCs given by intracranial injection, diminished the infarct volume and led to an improvement in behavioural indices of functional recovery. At molecular levels, the authors reported reduced levels of inflammatory markers IL-1β, IL-6, and TNF-α in MSCs-treated animals. Similarly, a reduced MMP-9 activity in polymorphonuclear cells in treated mice was associated with resistance to IgG leakages and loss of tight junction proteins [23].

### 2.15. Conditioned Medium from Adult Neural Progenitor Cells

Administration of stem cells at the lesion site is not necessary to achieve a therapeutic effect. Beneficial effects on tissue and behavioural recovery were reported after the administration of conditioned medium obtained from cultured stem cells. Further, hypoxic pre-conditioning enhances the production of factors, including IGF-1, BDNF, GDNF, VEGF, SDF-1, and HGF, that promote survival and plasticity [24,32,33,34,35,36,37,38].

For example, to assess whether secreted factors can mimic the neuroprotective and restorative effects of NPC, male C57BL6 mice were exposed to focal cerebral ischaemia and received intravenous treatment with a conditioned medium (CM) containing biologically active substances derived from cell cultures of neuronal progenitor cells from the subventricular zone up to 12 h after focal cerebral ischemia. The reduction in infarct volume was dose-dependent, as was brain leukocyte infiltration at 48 h post-administration, when the treatment. Neuroprotection persisted in the post-acute phase of stroke, producing an improved neurological recovery maintained throughout the observation period of 28 days. Long-term neuroprotection was associated with increased concentration of glial cell line-derived neurotrophic factor (GDNF) and vascular endothelial growth factor (VEGF) at 28 days, resulting in increased neurogenesis and angiogenesis. The observation that conditioned medium derived from the neural progenitor cell induce neuroprotection and neurological recovery suggests that cell transplantation can be dispensable by administering secreted factors [39].

### 2.16. Extracellular Vesicles Hypothesis

Extracellular vesicles (EVs) are 40–1000 nm nanosized vesicles that are released from many cell types into the extracellular space. Such vesicles are widely distributed in various body fluids and can readily cross body barriers, such as the blood brain barrier (BBB). The biogenesis of EV production starts from the plasma membrane invagination, which then furthers form inward buds forming so-called multivesicular bodies. During this process, several cellar containing molecules, such as mRNAs, miRNAs, proteins and metabolites are selectively packed into the vesicles. Thus, EVs can not only transfer biological messages between the cells in the body but also represent the physiological status of their parent cells, which makes EVs potential biomarker candidates [3]. Indeed, published literature has described that neuronal derived EVs can be isolated from plasma samples of patient and harness specific disease-related proteins, such as beta-amyloid and phosphorylated forms of tau in Alzheimer’s disease patients.

Remodelling of ischemic brain tissue involves interactions between neurons, glial and microvascular cells that create a microenvironment, in which neurological recovery may ensue [40]. The remodelling potential of the neurovascular unit serves as an important therapeutic target in stroke and other acute neurologic conditions.

Exosomes are released by virtually all cell types and have previously been shown to have restorative actions in the ischemic brain promoting neurological recovery and brain plasticity. In a head-to-head study, we have demonstrated that EVs obtained from mesenchymal stem cells (MSCs), which were intravenously administered 24 h after intraluminal middle cerebral artery occlusion (MCAO), promoted neurological recovery, long-term neuronal survival, angiogenesis, and neurogenesis very similar to MSCs, at the same time restoring post-ischemic immunodepression in peripheral blood [41]. These data indicated that EVs mediate the restorative effects of MSCs and that immune responses may be involved in EV actions, ie. EVs may have immune inhibitory or stimulatory activity which has profound consequence for their therapeutic actions [42,43,44,45,46].

Recent research has focused on the roles of exosomes, acting as bidirectional cargo between neurons, glia, vascular and perivascular cells, in brain homeostasis and plasticity [44,47]. The mechanisms by which exosomes exerts their neuroprotective effects are not precisely known. One study hypothesized that exosomes provide neuroprotection by promoting neuronal plasticity and increased angiogenesis in animals subjected to MCAo [46]. The hypothesis was tested in mouse model of MCAo subjected to post-stroke therapy with MSC-derived exosomes given by tail vein injection. Not surprinsingly, the MSC-derived exosomes improved the motor function and coordination ability which are associated with an improved recovery of the striatal structures. Moreover, the treatment had a pro-angiogenic effect which facilitated a vigorous neurogenic response to the striatal injury on a background of enhanced cerebral ischemia-associated immunosuppression [42].

### 2.17. Mitochondria Hypothesis

Brain recovery after an overt injury requires energy supply normally provided by the mitochondria. However, following brain injury, most of the cellular structures are destroyed, including the mitochondria. Therefore, one strategy to restore the energy supply is mitochondria transplantation which is supported by the ability of mitochondria to undergo mitochondrial fission and remodelling in response to higher metabolic demands. Therefore, mitochondria transfer is essential for a successful regenerative attempt of the damaged brain cellular structures [48]. In particular, the capacity of MSC to donate their mitochondria upon transplantation into the recipient cells of the damaged tissue proved to be one supportive mechanism which aid in restorative events in the brain, heart, and kidney [49]. In addition, the transplanted mitochondria aid in activating the innate immune response to the local injury by providing ROS, a crucial signalling mediator of inflammation [50]. 

The mitochondrial transfer is not unidirectional. Available data suggest that cues released by the damaged cells may trigger a bidirectional mitochondrial and mtDNA transfer between transplanted MSCs and the recipient’s cells. Thus, it could be that the transfer of mtDNA from the damaged cells to the transplanted MSCs may trigger mitophagy and as a consequence, more mitochondria are available to rescue the injured cells by providing more energy and a robust immune response within the damaged tissue [49,50]. 

The therapeutic potential of mitochondria transfer has been assessed in an experimental model of stroke. One study investigated the effect of the transfer of mitochondria from multipotent mesenchymal stem cells (MMSC) to astrocytes and neuron-like PC12 pheochromocytoma cells after lentivirus-driven increased expression of Miro1 in MMSC. Miro1 (Rho-GTPase 1, synonym: RhoT1), a calcium-sensitive adaptor protein which organize mitochondrial movement along microtubules inside the cells, was given intravenously to MCAo rats. The most important result of the transfer of mitochondria from the MMSC was the restoration of neural functions observed in vivo. More specifically, behavioural data analysis suggests a reduction of the neurological deficit of the upper limbs at 14 days post stroke in animals receiving intravenous injections of multipotent mesenchymal stem cells. Thus, it was concluded that the transfer of mitochondria is a very important component of the neuroprotective effect of MMSC after experimental ischemic stroke [50].

## 3. Conclusions

The field of stem cell therapy for cerebral ischemia has made significant progress in the last five years. Combination therapies in animal models include a mix of two or more therapeutic factors consisting of bone marrow stromal cells, exercise and thyroid hormones, endothelial progenitor cells and lentiviruses overexpressing the chemokine CXCL12. Mechanisms underlying the beneficial effects of transplanted cells include the “bystander” effects, paracrine mechanisms, or extracellular vesicles-mediated restorative effects. Mitochondria transfer also appears to be a powerful strategy for regenerative processes. We believe that the most promising stem cells for stroke therapy in animal models are the adipose tissue-derived mesenchymal stem cells (ADMSCs) which have demonstrated their therapeutic potential in stroke models by improving functional recovery, with an increase in markers related to brain repair.

We should not forget that there are also risks associated with the transplantation of stem cells into a lesioned brain [51]. Studies in humans are currently limited to small studies using autologous stem cells mainly aimed to assess tolerability and side-effects of human stem cells [52].

## Figures and Tables

**Table 1:** An overview of current stem cell therapies for cerebral ischemia.

Cell Source	Route	Timing	Subject	Effects	Reference
hAMSC	iTCX	24 h PS	MCAo, rat	Immunomodulatory function; improved behaviour	[4]
hAECs	iv	1.5-h; 1–3 days PS	MCAo, mice	Reduced inflammation, infarct volume and functional deficits.	[6]
hUCB-MSCs	tv	1-day PS	MCAo, rat	Prevented the induction of matrix metalloproteinases	[7]
hNPCs	iTCX	1-week PS	MCAo, rat	Improved functional results	[8,9]
hBMEPCs	iv	48-h PS		Improved angiogenesis	[10]
hAPOSCs	iTCX	1-hr PS	MCAo, mice	Decreased infarct volume; improved angiogenesis and functional results	[11]
hAPOSCs	iTCX	3–6 h PS	humans	No side effects after 12 mo	[11]
hiNSC		5-days PS	MCAo, pig	Good recovery of brain integrity, perfusion and function	[12,13]
hADMSCs	ia	1-h PS	MCAo, rat	Improved infarct volume	[21]
hEPC-cxcl12	iTCX	1-week PS	MCAo, mice	Improved angiogenesis, neurogenesis and functional results	[19]
rBM-MSCs	iv	2-h PS	MCAo, rat	Improved interhemispheric connectivity	[20]
H-rBMSCs	iTCX	1-h PS	MCAo, mice	Diminished infarct volumes, attenuated blood-brain barrier disruption	[23,24]
hADMSCs+Eenv	iv	2-days PS	MCAo, rat	Improved behavioural outcome	[22]
rBM-MSCs+ G-CSF	iv	3-h PS	MCAo, rat	Improved behavioural outcome	[25]
rBM-MNs+ G-CSF	iv	3-h PS	MCAo, rat	Improved behavioural outcome	[26]
MSCs+T3		24-h PS		Decreased infarct volume, decrease in apoptosis	[27]
MSC+PA		24-h PS		Decreased infarct volume, decrease in apoptosis	[27]

Abbreviations: hAMSC, human placenta amniotic membrane-derived mesenchymal stem cells; hAECs, human amnion epithelial cells; hUCB-MSCs, human umbilical cord blood-derived mesenchymal stem cells; hNPCs, human neuronal progenitor cells; hBMEPCs, bone marrow endothelial progenitor cells; hAPOSCs, adult human pluripotent-like olfactory stem cells; hiNSC, human induced pluripotent neural stem cells; hADMSCs, adipose human tissue-derived mesenchymal stem cells; hEPC-cxcl12, lentivirus overexpressing CXCL12 in human endothelial progenitor cells; rBM-MSCs, rat bone marrow-derived mesenchymal cells; H-rBMSCs, hypoxic preconditioning of rat BMSCs; iTCX, intracortical; iv, intravenously; tv, tail vein; ia, intra-arterial; PS, post stroke; PA, physical activity; T3, thyroid hormone; Eenv, enriched environment.
